# Association between Stages of Hepatic Steatosis and Physical Activity Performance in Adults with Metabolic Syndrome: A Cross-Sectional Analysis in FLIPAN Study

**DOI:** 10.3390/nu14091790

**Published:** 2022-04-24

**Authors:** Catalina M. Mascaró, Cristina Bouzas, Sofía Montemayor, Miguel Casares, Cristina Gómez, Lucía Ugarriza, Pere-Antoni Borràs, J. Alfredo Martínez, Josep A. Tur

**Affiliations:** 1 Research Group on Community Nutrition and Oxidative Stress, University of the Balearic Islands-IUNICS, 07122 Palma de Mallorca, Spain; c.mascaro@uib.es (C.M.M.); cristina.bouzas@uib.es (C.B.); sofiamf16@gmail.com (S.M.); cristina.gomez@ssib.es (C.G.); luciaugarriza@gmail.com (L.U.); 2 Health Institute of the Balearic Islands (IDISBA), 07120 Palma de Mallorca, Spain; 3 CIBEROBN (Physiopathology of Obesity and Nutrition CB12/03/30038), Instituto de Salud Carlos III (ISCIII), 28029 Madrid, Spain; 4 Radiodiagnosis Service, Red Asistencial Juaneda, 07011 Palma de Mallorca, Spain; casaresmiguel@gmail.com; 5 Clinical Analysis Service, University Hospital Son Espases, 07120 Palma de Mallorca, Spain; 6 Camp Redó Primary Health Care Center, 07010 Palma de Mallorca, Spain; 7 Area of Physical Education and Sports, Department of Pedagogy and Specific Didactics, University of the Balearic Islands, 07122 Palma de Mallorca, Spain; pa-borras@uib.es; 8 Cardiometabolics Precision Nutrition Program, IMDEA Food, CEI UAM-CSIC, 28049 Madrid, Spain; jalfredo.martinez@imdea.org; 9 Department of Nutrition, Food Sciences, and Physiology, University of Navarra, 31008 Pamplona, Spain

**Keywords:** physical activity, Mediterranean diet, Mediterranean lifestyle, non-alcoholic fatty liver disease, hepatic steatosis, fitness tests

## Abstract

Background: Non-alcoholic fatty liver disease (NAFLD) is the most common liver disease. The best treatment now is a healthy lifestyle with a Mediterranean diet and physical activity (PA). Objective: To assess the association between stages of hepatic steatosis and physical activity performance in adults with metabolic syndrome. Design: Cross-sectional study in 155 participants (40–60 years old) with MetS, a diagnosis of NAFLD by magnetic resonance imaging and BMI (body mass index) between 27 and 40 kg/m^2^. Methods: Stages of hepatic steatosis were assessed and defined according to the percentage of intrahepatic fat contents: stage 0 ≤ 6.4% (control group); stage 1 = 6.4–17.4%; stage 2 ≥ 17.4%. Fitness was assessed through ALPHA-FIT test battery for adults, aerobic capacity by Chester-step test and PA by accelerometry and Minnesota questionnaire. Results: Participants without NAFLD reported more years of education and major socioeconomic status than participants with NAFLD. A higher percentage of people in the most advanced stage of NAFLD were no smokers and no alcohol consumers. They also had higher stages of steatosis, lower sitting handgrip, standing handgrip, Chester step test values, sleep efficiency, and energy expenditure, and higher intensity of light and moderate physical activity, and self-reported physical activity. Conclusions: NAFLD patients showed lower fitness status, aerobic capacity, sleep efficiency and energy expenditure than non-NAFLD participants.

## 1. Introduction

Currently, the most common liver disease with a global prevalence greater than 25% is non-alcoholic fatty liver disease (NAFLD). It is the excessive accumulation of fat in liver, but without the abuse of alcohol consumption [1,2]. Additionally, it is defined as the impact of metabolic syndrome on hepatic metabolism. So, obesity, diabetes mellitus type 2 or high fasting glucose levels, hypertension and dyslipidemia are risk factors for NAFLD [3]. All of them can improve by changing lifestyle with diet and regular physical activity (PA). Lifestyle is the best existing treatment for NAFLD until now [4]. The most recommended diet is the Mediterranean diet, with principal intake are nuts, legumes, fruits, fish and olive oil, in contrast with a reduction of red and processed meats and sugars [5].

PA has a lot of benefits on health, such as reducing lipid disorders, weight, blood pressure, and important metabolic diseases such as diabetes mellitus type 2 and metabolic syndrome, all NAFLD-causing [6,7,8]. Additionally, regular PA improves cardiorespiratory fitness and decreases hepatic fat [9]. Low levels of aerobic fitness are associated with several health complications and premature deaths [10]. There is still little evidence, but regular PA and the consequent improvement of aerobic fitness, is related to a decreased risk of NAFLD [11]. 

Literature demonstrated that an active lifestyle with a Mediterranean diet and PA can improve metabolic syndrome characteristics and cardiovascular pathologies. So, a Mediterranean lifestyle reduces the severity and risk of NAFLD [12]. The role of PA on NAFLD is usually linked to a Mediterranean diet. There are only a few studies which assess an independent role of PA [13], suggesting that there can be a positive effect of PA on NAFLD even if there is not weight loss [13]. However, it is difficult to achieve changes in people’s unhealthy lifestyles. It is important that participants are motivated [14].

Information on assessing the interaction of PA and diet on NAFLD are scarce. Considering weight loss, the combination of PA and diet caused a higher weight loss than only diet [15]. Moreover, other literature showed that PA and diet resulted in a higher improvement of general NAFLD risk factors than PA or diet alone [16]. 

Recently, some manuscripts have been published showing the positive relationship between PA and NAFLD. Schneider et al. pointed out that increased PA is related to a decrease in NAFLD, even independent of adiposity [17]. Park et al. recently added that, in addition to regular PA practice, resistance training and high levels of PA decreases the risk of NAFLD [18]. Although there are more studies assessing these relationships, studies reporting the relationship between different PA parameters and NAFLD are still scarce. There is no clear association between fitness testing and NAFLD, nor between PA intensity and NAFLD. Existing literature indicated that the most appropriate intensities for benefits of PA on NAFLD are moderate to intense [19], but there is a lack of studies that go deeper into the topic. Most of the existing studies so far point to the benefits of PA for general health, but not for NAFLD. It seems to be a topic that is gradually emerging with more strength but remains little-known. The contribution of the present manuscript is to know, according to the stage of NAFLD, how good PA is. 

The aim of the present study was to assess the association between stages of hepatic steatosis and physical activity performance in adults with metabolic syndrome.

## 2. Methods

### 2.1. Design

This research was a cross-sectional analysis of baseline data from the clinical trial FLIPAN, an ongoing 2-year multicenter, parallel group and randomized trial. The FLIPAN trial was designed to assess the effect of nutritional intervention, based on a Mediterranean diet combined with personalized PA among patients with obesity and metabolic syndrome, with the objective of preventing and reversing NAFLD. The trial was registered at ClinicalTrials.gov (https://clinicaltrials.gov/ct2/show/NCT04442620; accessed on 25 February 2022) with number NCT04442620 [20].

### 2.2. Subjects, Recruitment, Intrahepatic Fat Contents Measurements, Randomization and Ethics

From June 2018 to January 2020, 237 people were contacted and assessed for screening. A total of 82 were excluded: 70 did not meet inclusion criteria and 12 declined their participation. Finally, the current clinical trial involves 155 participants aged 40–60 years, who were overweight or obese (body mass index between 27 and 40 kg/m^2^), and met at least three metabolic syndrome criteria according to the International Diabetes Federation (IDF) [21]. Abdominal magnetic resonance imaging was used to determine the intrahepatic fat contents (Signa Explorer 1.5T, General Electric Healthcare, Chicago, IL, USA) [22]. Three stages of hepatic steatosis were defined according to percentage of intrahepatic fat contents: S0 (stage 0 or control group) ≤ 6.4%; S1 (stage 1) = 6.4–17.4%; S2 (stage 2) ≥ 17.4% [23]. Participants were randomized equally into one of the three groups. Randomization was stratified by gender, type 2 diabetes mellitus and stage of steatosis. 

All participants were informed, and they provided written informed consent prior to participation. The study protocol and procedures were performed and approved according to the Declaration of Helsinki ethical standards and by the Ethics Committee of Research of Balearic Islands (ref. IB 2251/14 PI).

### 2.3. Fitness and Physical Activity Assessment

Fitness was determined through a battery of tests [24] such as balance, muscular strength, extensor power of the lower extremities, endurance of the trunk and upper extremities and general aerobic endurance. 

The physical condition of each individual was measured through six physical tests, four of which are detailed in “Fitness for Health: The ALPHA-FIT Test Battery for Adults 18–69” [24]. The first test (one-leg balance) was balancing on one leg for 60 s to measure the person’s postural control. It was performed on the dominant leg and there were two evaluations if the participant did not hold the maximum time. The second test (standing hand grip) was standing handgrip to measure the static force of the grip muscles. For this, a handgrip dynamometer was used (Takei TKK 5401, Tokyo, Japan, range = 5–100 kg, precision = 0.1 kg) and two evaluations were made with the dominant arm. The third test (sitting hand grip) was sitting handgrip, the same as above but seated. The fourth was jump-and-reach, consisted of taking a jump to measure the resistance and power of the lower extremities. The jump was measured with a ruler, in centimeters and 2 evaluations were made. The fifth test (modified push-up) was push-ups to evaluate the resistance of the trunk and upper extremities. A single evaluation was made on how many correct push-ups were done in 40 s. Finally, the sixth and last test was the Chester-Step to measure the aerobic capacity of individuals. For this, a 15 cm step was used (Chester Step Test Single Step 15 cm Height, Cartwright Fitness Limited, Huntington, Chester CH3 6DF, United Kingdom) and the rise and fall rhythm was marked with a metronome. Every two minutes the rhythm was increased until the five levels were completed or until 80% of the maximum heart rate was reached. Then, through the software, the maximum oxygen volume (VO_2_ max.) was calculated [25].

All tests were carried out on the same day and in the same order in which they are presented. They were performed by trained personnel and the best mark of each test was always taken. The results of each test were compared with the normal range of scores according to age and sex [24]. 

For one-leg balance, standing hand grip, jump-and-reach and modified push-up tests, normal range was set as the median of the population. Thus, a score below the normal range was defined below the median and a score above this range was defined above the median. The same was applied to the recording of accelerometer PA intensities: sedentary, light and moderate, as well as sleep efficiency.

Moreover, PA was measured by accelerometers (ActiGraph wGT3X-B; ActiGraph LLC, Pensacola, FL, USA) and reported by the Spanish version of Minnesota Leisure Time Physical Activity Questionnaire (mean weekly time). Measured and reported energy expenditure was presented as metabolic equivalent of task (MET)·minute/week [26,27]. Moreover, accelerometers measured sleep efficiency and different PA intensities: sedentary, light, moderate and vigorous in weekly minutes. 

The principle of actigraphy to measure sleep efficiency is the reduction of persons’ movements when sleeping. The accelerometer detects and records motor activity, generating rest-activity patterns that it transforms into sleep-wake cycles [28]. Computerized scoring algorithms automatically extract the data with software [29]. Sleep efficiency was defined as the ratio of time in minutes between the sleep onset and final awakening, i.e., which was spent asleep. The obtained value is expressed as a percentage of total time slept [30]. Subjects were considered to have a good sleep efficiency if above 85%, while passing the 90% threshold would be very good [31].

### 2.4. Physical Activity

From the one-leg balance, standing hand grip, jump-and-reach and modified push-up tests described in the previous section, a Functional Fitness Score has been developed. Thus, the physical parameters of the study are various: postural control, static force of the grip muscles, resistance and power of the lower extremities, resistance of the trunk and upper extremities. Together they contribute to knowing the global physical state of individuals under study and to offering alternatives to physical activity for a better health promotion [32]. Functional fitness score was based on a specific cut-off value for sex: the median of functional fitness tests (balance, standing handgrip, jump-and -reach, push-ups). Scores below the median were assigned a value of 0 points, which means that the individual’s functional fitness level was lower than the median for their sex. Scores above the median were assigned a value of 1 point, indicating that the functional fitness level of the individual was higher than the median for their sex. Thus, the best functional fitness level of the participant was 4, i.e., to obtain 1 point in each physical test. 

### 2.5. Other Health Outcomes

Information related to lifestyle and socioeconomic data were obtained by questionnaires: age, gender, education level, marital status, socioeconomic status according to job, smoking habit and alcohol consumption. Additionally, anthropometric measurements, dietary intake, adherence to the Mediterranean diet, blood samples and blood pressure were taken. 

### 2.6. Statistics

Analyses were performed with the SPSS statistical software package version 27.0 (SPSSS Inc., Chicago, IL, USA). Data are shown as median, interquartile range (IQR). Differences among groups were tested with Kruskal–Wallis analysis because variables did not follow normal distribution. Prevalence is expressed in sample size and percentage. Difference in prevalence’s among groups was tested using χ^2^ (all *p* values are two-tailed). Association between physical activity parameters (dependent variables) and stages of hepatic steatosis (independent variables) was analyzed by Odds Ratio (OR). For each item, 2 OR were calculated: crude and adjusted by sociodemographic characteristics (age, smoking habit, alcohol consumption and socioeconomic status according to job). All analysis were conducted for the whole sample. Results were considered statistically significant if *p*-value <0.05.

All variables that were not dichotomous were transformed taking the median as cutoff point.

## 3. Results

Sociodemographic characteristics according to stages of hepatic steatosis are available in Table 1. S2 participants were slightly younger (51 years) than S0 participants. In terms of years of education, S0 participants reported more years of education (18 years) than S1 and S2. Having a partner was more prevalent among three groups on the expense of being single, divorced, separated or widower, which was less prevalent. Even so, of the 3 groups, S1 was the one with the lowest percentage of married/unmarried partner people (68.1%). Tackling socioeconomic status according to job, the S0 control group had the highest percentage (25%) of people with a high level compared to the rest of the groups. On the other hand, the S2 group had a higher percentage of no smokers (97.1%) and no alcohol consumers (43.2%) than the S0 control group and S1 group. 

Table 2 shows physical activity parameters between participants with different stages of hepatic steatosis. People without NAFLD, the S0 control group, could make more correct modified push-ups (9 reps) than people in the S2 group. The same happened with sleep efficiency: people in the S0 control group had better sleep efficiency (93.8%) than participants in the S2 group. Tackling accelerometer data, S2 showed more sedentary intensity (654.8 min/day) than controls and S1. Regarding moderate intensity, people in the S1 group had slightly more minutes of this intensity (202.1 min/day) than people in the S0 control group, but this last group had more minutes of moderate intensity (201.3 min/day) than the S2 group. In terms of energy expenditure, controls and S2 group showed the same METs measured by the accelerometer (1.8 MET/day), whereas the S1 group showed more METs measured by the accelerometer (1.9 MET/day) than the S2 group. Additionally, S1 participants had major difference between measured and reported METs (1.7 MET/day) than S2 participants. Finally, controls obtained the best aerobic capacity or maximum oxygen uptake (36.7 mL O_2_/Kg/min) compared to S1 and S2. Additionally, S1 obtained more aerobic capacity (34.4 mL O_2_/Kg/min) than S2. Main differences between groups were observed in Chester step test, and accelerometry. Participants with higher intrahepatic fat contents showed lower aerobic capacity (Chester step test), higher sedentary activity, and lower moderate activity (by accelerometry).

Lastly, crude and adjusted OR for association between physical activity parameters and stages of hepatic steatosis is presented in Table 3. Control group or S0 group in the table (<6.4% of hepatic fat) was established as the reference. Crude and adjusted analysis showed that OR 0.44 (95% CI: 0.30–0.66)/0.41 (95% CI: 0.17–0.98) for S1 and OR 0.41 (95% CI: 0.26–0.65)/0.24 (95% CI: 0.09–0.63) for S2 were lower than S0 for sitting hand grip; and OR 0.24 (95% CI: 0.12–0.48)/OR 0.18 (95% CI: 0.07–0.49) for S1 and OR 0.11 (95% CI: 0.05–0.23)/OR 0.05 (95% CI:0.02–0.16) for S2 were lower than S0 for Chester-step. Standing hand grip item OR 0.40 (95% CI: 0.17–0.97) was lower than S0 only for S1 after adjustment. Additionally, for sleep efficiency, only adjusted analysis showed that OR 0.29 (95% CI: 0.10–0.85) for S1 and OR 0.13 (95% CI: 0.04–0.40) for S2 was lower than S0. On the other hand, S1 and S2 had a crude OR 7.14 (95% CI: 2.20–23.18) and OR 5.00 (95% CI: 1.39–17.94), respectively, higher than S0 for items regarding light and moderate intensity (S1 OR 4.00 (95% CI: 2.10–7.61) and S2 OR 3.30 (95% CI: 1.62–6.71)) of PA, but it disappeared after adjustment. Apart from that, S1 and S2 had the same crude OR for modified push-ups, it was 0.46 (95% CI: 0.26–0.81) lower than S0. After adjustment it disappeared for S1, but not for S2, it was 0.33 (95% CI: 0.11–0.98) lower than S0. Additionally, S1 and S2 had very similar crude OR for sedentary intensity of PA, it was 0.38 (95% CI: 0.20–0.69) lower than S0 for S1 and 0.34 (95% CI: 0.17–0.68) lower than S0 for S2. After adjustment it disappeared for S1, but it was 4.01 (95% CI: 1.28–12.55) higher than S0 for S2. Referent to energy expenditure, crude OR for measured accelerometer was 0.31 (95% CI: 0.16–0.62) lower than S0 for S2. After adjustment, also it was 0.31 (95% CI: 0.10–0.91) lower than S0 but for S1, while it was 0.06 (95% CI: 0.02–0.24) lower than S0 for S2. Reported Minnesota had a crude OR 0.51 (95% CI: 0.34–0.75) lower than S0, and 0.27 (95% CI: 0.11–0.67) lower than S0 after adjustment for S1. 

## 4. Discussion

Current findings showed that fitness tests, aerobic capacity, sleep efficiency and energy expenditure were lower in NAFLD patients than in non-NAFLD participants. Previous studies reported that there were not differences between hand grip strength in standing or sitting position [33,34]. Literature also showed that lower muscular strength is associated with metabolic syndrome components [35]. Accordingly, in the current study the lower sitting hand grip strength was more likely in people with advanced stages of intrahepatic steatosis (S1 and S2). After adjustment by confounders, this evidence was the same for standing hand grip and people with intermediate stage hepatic steatosis (S1). 

It was previously showed that resistance exercise based on push-ups improve metabolic syndrome components in subjects with NAFLD [36]. However, it was showed that obesity decreases maximal muscle strength and difficulties some exercises [37]. Current results reported that correct modified push-ups are less likely in subjects with advanced NAFLD than subjects without NAFLD (stage 0) or less advanced stages (S1 after adjustment by confounders). It reaffirmed that excess fat interferes with strength and exercise performance, as mentioned above.

Previous literature pointed out that high intensity intervals or moderate intensity continuous training can reduce visceral lipids and intrahepatic triglycerides in diabetic obese people with NAFLD [38]. Additionally, aerobic training helps to improve other parameters related to NAFLD such as levels of high-density lipoprotein (HDL) and low-density lipoprotein (LDL) cholesterol, total cholesterol and/or aspartate aminotransferase (AST) and alanine aminotransferase (ALT) [39]. However, in the current study, it was shown that people with NAFLD had more difficulty performing these PA practices. People with moderate hepatic steatosis (S1) had lower aerobic capacity from Chester-step than people without NAFLD (S0 or control group). Looking people with hepatic steatosis S2 in contrast with controls, the aerobic capacity was even lower, which was consistent with existing literature [40]. Previous literature also reported that people with high a lack of PA showed health problems, such as a weaker heart, slower metabolism, poorer fitness or reduced aerobic capacity, among others [41] which agrees with the current results. Available literature on PA intensity pointed out that most subjects with NAFLD showed PA sedentary level [42]. However, the current results were not consistent with this evidence. Subjects with advanced stages of hepatic steatosis (both S1 and S2) were less likely to be sedentary than those without fatty liver. Only after adjustment by sociodemographic characteristics, people with S2 showed more likely to be sedentary than controls without NAFLD. Participants with S1 or S2 were more likely to practice light and moderate levels of PA than participants without NAFLD. In contrast, nobody registered vigorous levels of PA. Socioeconomic status also influences dietary habits, time and interest in PA [1,43]. Accordingly, the explanation to current findings may be that people who are aware of their disease try to practice more PA (minutes/day) to improve their health than people without disease. In relation to these results and in favor of PA practice, there is a study that demonstrated how increased moderate/vigorous PA is necessary for prevention of NAFLD [19]. However, as mentioned above, the participants in this study did not record vigorous intensity levels.

Current results reported that patients with S1 and S2 were more likely to have lower sleep efficiency than patients without fatty liver disease. Inadequate sleep was associated with development and/or progression of NAFLD, but also the liver alteration affects quality of sleep [44]. In adults, short sleep duration and low sleep efficiency were risk factors for NAFLD [45]. 

Using PA Minnesota questionnaire, current results showed a certain change in people with advanced stages of NAFLD, with a higher probability of having light and/or moderate PA intensities, and less sedentary, in contrast with people without NAFLD; the truth is that regular PA is a pending subject in general society [46]. In terms of energy expenditure, current study reported lower METs measured by accelerometer was more likely in people with S2 group than people in S0 control group. Moreover, lower METs reported by Minnesota questionnaire was more likely in people with S1 than people in S0 control group. In both cases, general probability of having lower METs was shown in advanced stages of hepatic steatosis with respect to those without NAFLD. Current results were consistent with the existing literature. Patients with NAFLD exhibited lower METs than patients without NAFLD [45]. Moreover, adults with most severe metabolic syndrome and its complications had lower vigorous PA levels and reduced METs per day [47]. Leisure-time PA is a protective factor for NAFLD, whereas sedentarism is a risk factor, but existing literature confirmed that the tendency of the actual population is to have a sedentary lifestyle. That is why changes in lifestyle are so important as a treatment or prevention of NAFLD [48].

Current findings also reported that S0 control group had the highest percentage of people with high level of socioeconomic status compared to S1 and S2 groups. Additionally, people in the S0 control group had more years of education than people in the S1 and S2 groups. The literature showed that better socioeconomic status in families with children was related to a correct dietary pattern, lower sugar consumption, reduced insulin resistance or diabetes mellitus type 2 and better sleep efficiency. When these risk factors for NAFLD improved, stages of hepatic steatosis also could improve [4]. 

Current results also reported that people in the S2 group had a higher percentage of no smokers and no alcohol consumers than the better steatosis participants (controls without steatosis and S1 group), reaffirming the idea that they were trying to improve their lifestyle habits avoiding to worse NAFLD. It is well known that smoking is a risk factor for NAFLD development [49], as is alcohol consumption, even if it is a light drinking habit [50]. Lifestyle factors, such as diet composition, PA practice, and/or moderate alcohol consumption influence NAFLD progression [51]. Most individuals with metabolic risk expressed high interest to learn about the topic and how they could improve their NAFLD status [52]. 

### Strengths and Limitations of the Study

The present study contributes to the very limited evidence tackling the association between individual motor fitness tests and adult NAFLD population. There is some evidence linking some PA tests to metabolic syndrome, but there is little information about this topic too. Other strengths of the present study include its large proposal of PA parameters (such as motor fitness tests, aerobic capacity, PA intensity and energy expenditure) and the relationship of the topic with sociodemographic and socioeconomic to obtain a major vision. On the top of strengths, results would be very easily implemented into clinical practice, as treatment of NAFLD. This is important because a pharmacological treatment for NAFLD does not exist [53]. 

However, the current study has limitations. The main limitation was the small sample size as well as some missing values. Another important limitation would be that causal inferences cannot be established, because it has a cross-sectional design. Secondly, the Minnesota questionnaire, even after being validated, might overestimate total daily METs and, therefore, the real reported energy expenditure. Thirdly, the physical condition of patients was not always ideal to perform the tests presented correctly, which could interfere with the final results. Lastly, all participants in the present study were between 40–60 years old and about to start a healthier lifestyle as part of FLIPAN trial, and also had metabolic syndrome, which is a limitation to make these results extensible to general adult population.

## 5. Conclusions

The current study reported that lower fitness status, aerobic capacity, sleep efficiency and energy expenditure were shown in NAFLD patients. Accordingly, subjects with NAFLD need to modify their lifestyle, following a healthy diet and practicing daily PA, to maintain their physical fitness as a contributor to their health status.

## Figures and Tables

**Table 1:** Sociodemographic characteristics according to stages of hepatic steatosis.

	S0 (*n* = 41)	S1 (*n* = 72)	S2 (*n* = 37)	
	Median (IQR)	Median (IQR)	Median (IQR)	*p*
Age (years)	52.0 (12.0) ^b^	53.0 (12.0)	51.0 (9.0) ^b^	0.023
Education (years)	18.0 (7.0) ^a,b^	14.5 (6.0) ^a^	15.0 (7.0) ^b^	<0.001
	*n* (%)	*n* (%)	*n* (%)	
Gender				0.279
Female	18 (43.9)	28 (38.9)	13 (35.1)	
Male	23 (56.1)	44 (61.1)	24 (64.9)	
Marital status				<0.001
Single	1 (2.4)	11 (15.3)	2 (5.4)	
Married/unmarried partner	34 (82.9)	49 (68.1)	30 (81.1)	
Divorced/separated	6 (14.6)	11 (15.3)	5 (13.5)	
Widower	0 (0)	1 (1.4)	0 (0)	
Socioeconomic status according job				<0.001
Low	4 (50.0)	29 (69.0)	21 (80.8)	
Medium	2 (25.0)	12 (28.6)	4 (15.4)	
High	2 (25.0)	1 (2.4)	1 (3.8)	
Smoking habit				<0.001
No	34 (85.0)	57 (81.4)	34 (97.1)	
≥1 cigarette/day	6 (15.0)	13 (18.6)	1 (2.9)	
Alcohol consumption				<0.001
No	5 (12.2)	18 (25.0)	16 (43.2)	
Yes, <7 drinks/week	29 (70.7)	40 (55.6)	17 (45.9)	
≥7 drinks/week	7 (17.1)	14 (19.4)	4 (10.8)	

Abbreviations: S0 = stage 0, S1 = stage 1, S2 = stage 2. Stages of hepatic steatosis are classified according to percentage of hepatic fat: S0: <6.4% (control group without NAFLD); S1: 6.4–17.4%; S2: >17.4%. Difference in means between groups were tested by Kruskal–Wallis test (no normally distributed). Differences in prevalence’s across groups were examined using χ^2^. Different letters indicate statistically significant differences between groups (a, b) according post hoc analysis, with a *p*-value < 0.05. Number of missing values per parameter are: age = no missing values; education years = no missing values; gender = no missing values = marital status = no missing values; socioeconomic status according job = 3 missing values in S0/3 missing values in S1/1 missing values in S2; smoking habit = 1 missing value in S0/2 missing values in S1/2 missing values in S2; alcohol consumption = no missing values.

**Table 2:** Physical activity parameters between participants with different stages of hepatic steatosis.

	S0	S1	S2	
	Median (IQR)	Median (IQR)	Median (IQR)	*p* Value
Motor fitness tests				
One-leg balance (s)	52.0 (15.1)	60.0 (27.7)	32.7 (46.0)	0.110
Standing handgrip (kg)	37.2 (23.2)	40.6 (23.5)	39.1 (15.3)	0.275
Jump-and-reach (cm)	26.0 (18.0)	23.0 (10.0)	20.0 (12.0)	0.154
Modified push-up (reps)	9.0 (6.0) ^b^	10.0 (7.0)	7.0 (6.0) ^b^	0.036
Fitness score tests	2.0 (4.0)	2.0 (3.0)	2.0 (1.0)	0.668
Sitting handgrip (kg)	36.0 (25.0)	39.9 (22.6)	35.0 (19.0)	0.090
Chester-step (ml O_2_/kg/min)	36.7 (11.2) ^a,b^	34.4 (10.4) ^a,c^	28.3 (9.5) ^b,c^	<0.001
Intensity PA (accelerometer)				
Sedentary (min/day)	648.1 (120.9) ^b^	610.4 (157.9) ^c^	654.8 (142.4) ^b,c^	0.003
Light (min/day)	528.4 (118.4)	491.9 (174.4)	541.7 (122.9)	0.188
Moderate (min/day)	201.3 (168.6) ^a,b^	202.1 (50.7) ^a^	160.9 (81.1) ^b^	<0.001
Sleep efficiency (%)	93.8 (3.3) ^b^	92.5 (6.7)	91.5 (3.3) ^b^	0.007
Vigorous (min/day)	0	0	0	1.000
Energy expenditure				
Measured accelerometer (MET/day)	1.8 (0.5) ^b^	1.9 (0.3) ^c^	1.8 (0.3) ^b,c^	<0.001
Reported Minnesota (MET/day)	0.3 (0.5)	0.3 (0.4)	0.3 (0.4)	0.188
Measured-Reported (MET/day)	1.6 (0.8)	1.7 (0.5) ^c^	1.5 (0.2) ^c^	0.009

Abbreviations: cm = centimeter, Kg = kilogram, MET = metabolic equivalents of task, min = minutes, ml = milliliter, No. = number; O_2_ = oxygen, PA = physical activity, reps = repetitions, s = seconds, S0 = stage 0, S1 = stage 1, S2 = stage 2. Stages of hepatic steatosis are classified according to percentage of hepatic fat: S0: <6.4% (control group without NAFLD); S1: 6.4–17.4%; S2: >17.4%. Differences in means between groups were tested by Kruskal–Wallis test (no normally distributed). Different letters indicate statistically significant differences between groups (a, b, c) according post hoc analysis, with a *p*-value < 0.05. Number of missing values per parameter are: one-leg balance = 3 missing values in S0/3 missing values in S1/1 missing values in S2; standing handgrip = 3 missing values in S0/3 missing values in S1/1 missing values in S2; jump-and-reach = 3 missing values in S0/3 missing values in S1/1 missing values in S2; modified push-up = 2 missing values in S0/2 missing values in S1/7 missing values in S2; Fitness score tests = 3 missing values in S0/4 missing values in S1/1 missing values in S2; sitting handgrip = 1 missing value in S0/1 missing value in S1/no missing values in S2; Chester-step = 2 missing values in S0/2 missing values in S1/7 missing values in S2; sedentary = 2 missing values in S0/3 missing values in S1/1 missing values in S2; light = 2 missing values in S0/3 missing values in S1/1 missing values in S2; moderate = 2 missing values in S0/3 missing values in S1/1 missing values in S2; sleep efficiency = 3 missing values in S0/4 missing values in S1/1 missing values in S2; vigorous = 2 missing values in S0/3 missing values in S1/1 missing values in S2; measured accelerometer = 2 missing values in S0/3 missing values in S1/1 missing values in S2; reported Minnesota = no missing values in S0/2 missing values in S1/1 missing value in S2; measured-reported = 2 missing values in S0/3 missing values in S1/1 missing values in S2.

**Table 3:** Association between physical activity parameters (dependent variables) and stages of hepatic steatosis (independent variables).

		S0	S1	S2
		OR (95% CI)	OR (95% CI)	OR (95% CI)
Motor fitness tests				
One-leg balance	Crude OR	1.00 (ref.)	0.61 (0.25–1.50)	0.46 (0.17–1.21)
	OR Adjusted 1	1.00 (ref.)	0.83 (0.31–2.19)	0.56 (0.19–1.64)
Standing handgrip	Crude OR	1.00 (ref.)	0.65 (0.29–1.46)	0.75 (0.32–1.74)
	OR Adjusted 1	1.00 (ref.)	0.40 (0.17–0.97) *	0.39 (0.15–1.00)
Jump-and-reach	Crude OR	1.00 (ref.)	1.27 (0.56–2.85)	0.98 (0.42–2.27)
	OR Adjusted 1	1.00 (ref.)	0.71 (0.29–1.75)	0.60 (0.23–1.57)
Modified push-up	Crude OR	1.00 (ref.)	0.46 (0.26–0.81) *	0.46 (0.25–0.85) *
	OR Adjusted 1	1.00 (ref.)	0.44 (0.16–1.22)	0.33 (0.11–0.98) *
Fitness score tests	Crude OR	1.00 (ref.)	0.56 (0.25–1.29)	0.49 (0.21–1.17)
	OR Adjusted 1	1.00 (ref.)	0.41 (0.14–1.17)	0.34 (0.11–1.04)
Sitting handgrip	Crude OR	1.00 (ref.)	0.44 (0.30–0.66) *	0.41 (0.26–0.65) *
	OR Adjusted 1	1.00 (ref.)	0.41 (0.17–0.98) *	0.24 (0.09–0.63) *
Chester-step	Crude OR	1.00 (ref.)	0.24 (0.12–0.48) *	0.11 (0.05–0.23) *
	OR Adjusted 1	1.00 (ref.)	0.18 (0.07–0.49) *	0.05 (0.02–0.16) *
Intensity PA (accelerometer)				
Sedentary	Crude OR	1.00 (ref.)	0.38 (0.20–0.69) *	0.34 (0.17–0.68) *
	OR Adjusted 1	1.00 (ref.)	1.88 (0.65–5.47)	4.01 (1.28–12.55) *
Light	Crude OR	1.00 (ref.)	7.14 (2.20–23.18) *	5.00 (1.39–17.94) *
	OR Adjusted 1	1.00 (ref.)	1.21 (0.18–8.15)	1.74 (0.24–12.65)
Moderate	Crude OR	1.00 (ref.)	4.00 (2.10–7.61) *	3.30 (1.62–6.71) *
	OR Adjusted 1	1.00 (ref.)	1.97 (0.72–5.42)	0.53 (0.18–1.54)
Sleep efficiency	Crude OR	1.00 (ref.)	0.96 (0.53–1.74)	0.54 (0.28–1.05)
	OR Adjusted 1	1.00 (ref.)	0.29 (0.10–0.85) *	0.13 (0.04–0.40) *
Energy expenditure				
Measured accelerometer	Crude OR	1.00 (ref.)	1.04 (0.58–1.85)	0.31 (0.16–0.62) *
	OR Adjusted 1	1.00 (ref.)	0.31 (0.10–0.91) *	0.06 (0.02–0.24) *
Reported Minnesota	Crude OR	1.00 (ref.)	0.51 (0.34–0.75) *	0.64 (0.41–1.01)
	OR Adjusted 1	1.00 (ref.)	0.27 (0.11–0.67) *	0.41 (0.16–1.09)
Measured-Reported	Crude OR	1.00 (ref.)	0.82 (0.46–1.46)	0.58 (0.30–1.12)
	OR Adjusted 1	1.00 (ref.)	1.42 (0.52–3.92)	0.63 (0.22–1.83)

Abbreviations: OR. Odds Ratio. *OR adjusted 1*: Odds Ratio adjusted by sociodemographic characteristics (age, smoking habit, alcohol consumption and socioeconomic status according to job), S0 = stage 0, S1 = stage 1, S2 = stage 2. Stages of hepatic steatosis are classified according to percentage of hepatic fat: S0: <6.4% (control group without NAFLD); S1: 6.4–17.4%; S2: >17.4%. * *p* < 0.05 vs reference (1.00).

## Data Availability

There are restrictions on the availability of data for this trial, due to the signed consent agreements around data sharing, which only allow access to external researchers for studies following the project purposes. Requestors wishing to access the trial data used in this study can make a request to pep.tur@uib.es.

## Abbreviations

ALT: alanine aminotransferase; AST: aspartate aminotransferase; HDL-cholesterol: high-density lipoproteins cholesterol; IDF: International Diabetes Federation; IQR: interquartile range; LDL-cholesterol: low-density lipoproteins cholesterol; MET: metabolic equivalent of task; NAFLD: non-alcoholic fatty liver disease; OR: Odds Ratio; PA: Physical activity; S0: stage of hepatic steatosis 0 or control group (≤6.4% hepatic fat); S1: stage of hepatic steatosis 1 (6.4–17.4% hepatic fat); S2: stage of hepatic steatosis 2 (≥17.4% hepatic fat).
